# Effects of Rice-Field and Pond Culture Systems on the Nutritional Quality, Metabolomic Profiles and Intestinal Microbiota of Largemouth Bass (*Micropterus salmoides*)

**DOI:** 10.3390/foods15193369

**Published:** 2026-09-22

**Authors:** Linjun Zhou, Rui Jia, Kepeng Wei, Qiqin Yang, Yiran Hou, Chengfeng Zhang, Bing Li, Jian Zhu

**Affiliations:** 1 Key Laboratory of Integrated Rice-Fish Farming Ecology, Ministry of Agriculture and Rural Affairs, Freshwater Fisheries Research Center, Chinese Academy of Fishery Sciences, Wuxi 214081, China; zhoulinjun@ffrc.cn (L.Z.);; 2 Wuxi Fisheries College, Nanjing Agricultural University, Wuxi 214081, China; 3 Shandong Huajian Inspection Co., Ltd., Linyi 276000, China; 4 Fishery Technology Extension Station of Yunnan, Kunming 650034, China

**Keywords:** culture modes, rice-fish co-culture, pond culture, amino acid metabolism, fatty acid composition, *Micropterus salmoides*

## Abstract

As aquaculture production becomes increasingly quality-oriented, understanding how culture systems affect the nutritional characteristics of edible fish muscle is important for evaluating aquaculture products. In this study, growth performance, muscle amino acid and fatty acid composition, metabolomic profiles and intestinal microbiota were compared between largemouth bass (*Micropterus salmoides*) reared in a rice-field culture system (MR) and a conventional pond system (MP). Overall growth performance was comparable between the two systems, although MR fish exhibited a higher condition factor. Total hydrolyzed amino acid contents were comparable between the groups, whereas differences in several free amino acids suggested distinct potential taste-related characteristics of the edible muscle. MP fish contained higher amounts of total fatty acids, saturated fatty acids, monounsaturated fatty acids, polyunsaturated fatty acids, n-3 PUFA, EPA and DHA, whereas MR fish exhibited a higher n-3/n-6 PUFA ratio. Muscle metabolomic profiles also differed between the two systems, particularly for metabolites associated with amino acid, purine and lipid metabolism. In addition, MR and MP fish exhibited distinct intestinal microbial communities, with higher microbial richness in MR fish and different dominant bacterial taxa between the systems. Overall, the rice-field culture system represents a promising ecological alternative for largemouth bass production because it maintained growth performance comparable to pond culture while producing a distinct edible-muscle nutritional profile, including a higher n-3/n-6 PUFA ratio. Pond-cultured fish, however, contained higher absolute amounts of several nutritionally important fatty acids, including n-3 PUFA, EPA and DHA. The distinct intestinal microbial communities observed between the systems further indicate different gut ecological responses to the two culture environments.

## 1. Introduction

As aquaculture transitions from yield-driven expansion to green, efficient and quality-oriented production, understanding how culture systems shape fish growth performances and nutritional quality has become increasingly important [1,2]. Culture systems differ in water environmental conditions, feed utilization patterns and available activity space, while also reshaping nutrient sources, microbial exposure and ecological interactions within the farming environment [3,4,5]. Although conventional pond culture is easily managed and provides relatively stable yields as well as intensive feed inputs, but the environmental pressures under high-density production may destabilize water quality and influence fish metabolism and product quality [6,7]. In contrast, rice-fish integrated farming is increasingly recognized as a sustainable aquaculture model because it promotes nutrient recycling and ecological interactions between rice and fish, thereby improving resource-use efficiency and reducing external inputs in paddy ecosystems [8,9]. For instance, rice plants in rice-fish systems can assimilate nitrogen and phosphorus from the water and thereby improve the culture environment, whereas fish promote internal nutrient cycling through the consumption of weeds, insects and other natural food resources [10,11]. Despite these advantages, the effects of different culture systems on muscle quality formation in carnivorous fish remain inconsistent. Rice-fish systems may improve muscle nutrition and flavor quality by providing more diverse natural food resources and a more complex microecological environment. A study on *Opsariichthys bidens* reported that rice-field culture has been shown to increase muscle protein content, enrich flavor-related compounds, and enhance the combined levels of DHA and EPA compared with pond culture [12]. In contrast, pond culture may favor lipid deposition and energy storage through more stable feed supply and more controllable growth conditions [4]. He et al. found that *Oreochromis niloticus* reared in pond tended to exhibit higher muscle lipid accumulation than those cultured in ecological rice-field systems [5]. For carnivorous fish in particular, their greater dependence on high-protein diets and lipid nutrition means that variation in culture environment and feed inputs may simultaneously influence endogenous metabolic activity, energy allocation and nutrient deposition [13]. These uncertainties highlight the need to compare the effects of different culture systems on fish muscle quality formation from multiple perspectives.

Largemouth bass (*Micropterus salmoides*), a freshwater fish native to North America, has become a globally farmed freshwater fish of high economic value, valued for its rapid growth, tender flesh and strong consumer acceptance [14,15]. Traditional rice–fish farming has historically relied mainly on cyprinid and other omnivorous species adapted to shallow paddy environments, whereas the introduction of largemouth bass represents a relatively recent extension toward high-value carnivorous fish [16]. *M. salmoides* has relatively high dietary protein requirements and depends strongly on formulated feeds, making its adaptation to the spatially heterogeneous rice-field environment less straightforward than that of traditional rice–fish species [17]. Compared with conventional pond culture, rice-field systems integrate fish and crop production and provide opportunities for nutrient recycling, ecological interactions and complementary use of natural food resources. Therefore, incorporating largemouth bass into rice fields may broaden the species and product portfolio of integrated aquaculture while maintaining the land-sharing advantages of rice–fish production [18]. Published evidence on rice–largemouth bass culture remains limited. Our previous study demonstrated that *M. salmoides* can be reared in integrated rice–fish systems, although stocking density markedly affected its growth and physiological status [19]. Nevertheless, its growth performance and edible muscle nutritional quality relative to conventional pond culture remain insufficiently understood. More broadly, previous studies on *M. salmoides* have mainly focused on feed nutrition, lipid metabolism and health regulation [20,21], whereas comparatively less attention has been paid to how alternative production systems influence the nutritional quality of its edible muscle. Fish flesh quality is a multidimensional trait that can be evaluated through nutritional composition and the biochemical state of edible muscle. Amino acids and fatty acids directly determine important nutritional characteristics of muscle, whereas metabolomic profiling can provide complementary information on biochemical processes associated with nutrient turnover and energy metabolism, in parallel, the intestinal microbiota is responsive to diet, culture environment, and host physiological status and can therefore provide additional information on the biological responses of fish to different production systems [22,23,24]. Accordingly, these complementary measurements can characterize both the nutritional properties of the edible product and broader biological responses of fish to different culture environments.

Here, we asked whether rice-field and conventional pond culture systems produce differences in the nutritional characteristics of edible largemouth bass muscle and whether these differences are accompanied by distinct muscle metabolomic and intestinal microbial profiles. Based on previous evidence that culture environment can influence nutrient availability, activity patterns and microbial exposure, we hypothesized that the two systems would maintain broadly comparable growth performance but would differ in muscle nutritional composition, particularly in free amino acid and fatty acid profiles. We further hypothesized that the two culture systems would be associated with distinct muscle metabolomic and intestinal microbial characteristics. To test these hypotheses, we compared growth performance, muscle amino acid and fatty acid composition, untargeted muscle metabolomics and intestinal microbial communities between rice-field and pond-cultured fish. This study was intended to provide information relevant to quality-oriented aquaculture and the nutritional evaluation of edible largemouth bass.

## 2. Materials and Methods

### 2.1. Experimental Design and Sample Collection

The experiment was conducted at the Jingjiang Experimental Base (32°08′ N, 120°19′ E) of the Freshwater Fisheries Research Center, located in a subtropical monsoon climate zone with an annual precipitation of approximately 1078 mm and an annual mean temperature of 16–20 °C. All largemouth bass juveniles used in this study were bred at the Jingjiang Experimental Base. Before the trial, individuals of similar body size were selected, weighed, and randomly allocated to either the rice–fish co-culture system (MR) or the conventional pond system (MP). The rice–fish system consisted of a 1624 m^2^ rice-growing area with a water depth of approximately 0.20 m and a 176 m^2^ peripheral fish trench with a depth of approximately 0.80 m. Water exchanged freely between the rice-growing area and the surrounding ditch, while largemouth bass were restricted to the peripheral ditch by barriers. Rice seedlings (Nangeng 5055) were transplanted in mid-June 2021 and harvested in early November 2021. Largemouth bass juveniles were stocked on 30 July 2021 and harvested on 8 October 2021. Rice-field management followed conventional local agricultural practices [25]. Each pond in the conventional pond system had an area of 2664 m^2^ and a water depth of approximately 2.0 m. When an obvious decline in water level was observed in MP or MR system, water from the adjacent reservoir was supplied to restore the culture water level. Each culture system included three biological replicates, and the same stocking density (120 g m^−3^) was maintained across all experimental units. The mean initial body weight of the fish was 70.02 ± 2.30 g. Fish were fed the same commercial pelleted diet (Changzhou Haida Biological Feed Co., Ltd., Changzhou, China), containing 47% crude protein, 5% crude lipid, 18.0% crude ash, 3.0% crude fiber, 10% moisture, 1.2% phosphorus, and 2.7% lysine. Fish were fed twice daily at approximately 08:00 and 17:00, and the daily feed ration (approximately 2–3% of body weight) was adjusted according to fish biomass.

At the end of the 10-week culture period, 120 fish from each group were randomly sampled for measurements of body weight, body length, and other growth-related indices. Subsequently, 36 fish from each group were randomly selected and anesthetized with 50 mg L^−1^ MS-222 (Sigma-Aldrich, St. Louis, MO, USA), after which muscle and intestinal tissues were collected for subsequent analyses. All experimental procedures were conducted in accordance with animal-welfare requirements and were approved by the Freshwater Fisheries Research Center.

### 2.2. Determination of Amino Acid Composition in Muscle Tissue of M. salmoides

Hydrolyzed amino acids were determined following previously described methods with minor modifications [26]. Briefly, approximately 100 mg of each sample was mixed with 10–15 mL of 6 mol L^−1^ hydrochloric acid. The mixture was cooled in a freezing agent for 3–5 min, flushed with nitrogen, sealed and hydrolyzed at 110 ± 1 °C for 22 h in a thermostatically controlled drying oven. After hydrolysis, the tube was cooled to room temperature and the hydrolysate was filtered into a 50 mL volumetric flask. The hydrolysis tube was rinsed several times with a small volume of water, and the rinses were combined with the filtrate. The solution was then brought to volume with water and mixed thoroughly. A 1.0 mL aliquot of the filtrate was transferred to a 15 mL tube and dried under reduced pressure at 40 °C. The residue was reconstituted in 1.0 mL of sodium citrate buffer (pH 2.2), mixed thoroughly and filtered through a 0.22 μm membrane before amino acid analysis using an automatic amino acid analyzer.

Free amino acids were determined according to the method described by Luo et al. [27]. About 100 mg sample was weighed into a 10 mL centrifuge tube, dissolved in 0.02 mol L^−1^ hydrochloric acid and brought to volume. A C18 solid-phase extraction column was preconditioned with 5 mL of methanol followed by 5 mL of water. Subsequently, 2.5 mL of the sample extract was loaded onto the column, followed by 1.5 mL of 0.02 mol L^−1^ hydrochloric acid. The eluate was collected, adjusted to 5 mL with 0.02 mol L^−1^ hydrochloric acid and filtered through a 0.45 μm membrane and were then determined using an automatic amino acid analyzer (Hitachi LA8080, Hitachi, Tokyo, Japan). Separation was performed on a sulfonic acid cation-exchange resin column, with detection at 570 and 440 nm. The injection volume was 20 μL, and the reaction temperature was maintained at 135 ± 5 °C. Free arginine was below the detection limit of the analytical method and therefore could not be reliably quantified. Accordingly, it was not included in the quantitative free-amino-acid dataset or in the calculation of total free amino acids (TFAA).

### 2.3. Determination of Fatty Acid Composition in Muscle Tissue of M. salmoides

Muscle fatty acid composition was determined following a previously described method [28]. Approximately 500 mg of freeze-dried muscle was weighed and subjected to acid hydrolysis with 8.3 mol L^−1^ HCl at 70–80 °C for 40 min. After hydrolysis, lipids were recovered from the resulting digest using a 1:1 (*v*/*v*) mixture of diethyl ether and petroleum ether. The extracted lipid fraction was then converted to fatty acid methyl esters (FAMEs). Briefly, 8 mL of 2% sodium hydroxide in methanol was added to the lipid extract, and the mixture was refluxed in an 80 ± 1 °C water bath until no visible oil droplets remained. Subsequently, 7 mL of 15% boron trifluoride in methanol was added through the condenser, and the reaction was continued for a further 2 min under the same temperature conditions. After methylation, the apparatus was rinsed with a small amount of water, and the reaction mixture was cooled to room temperature. FAMEs were extracted by adding 20 mL of n-heptane, followed by vigorous mixing for 2 min. Saturated sodium chloride solution was then added to facilitate phase separation. Approximately 5 mL of the upper n-heptane phase was collected and transferred to a 25 mL tube containing approximately 4 g of anhydrous sodium sulfate. After shaking for 1 min and standing for 5 min, the clarified supernatant was transferred to a chromatographic vial for analysis. FAMEs were separated and quantified using an Agilent 6890 gas chromatograph equipped with a flame ionization detector (FID) and an HP-88 capillary column (100 m × 0.25 mm i.d., 0.20 μm film thickness; Agilent Technologies, Santa Clara, CA, USA). The injector and detector temperatures were maintained at 250 and 260 °C, respectively. Nitrogen was used as the carrier gas at a constant flow rate of 1.0 mL min^−1^, and samples were introduced using a split injection mode with a split ratio of 40:1. Individual fatty acids were identified by comparing their retention times with those of a commercial FAME standard mixture (Sigma-Aldrich, St. Louis, MO, USA).

### 2.4. Analysis of Differential Metabolites of M. salmoides Muscle

Untargeted metabolomic analysis was conducted using a standardized LC–MS workflow provided by Novogene Co., Ltd. (Beijing, China). Briefly, 100 mg of tissue sample was ground in liquid nitrogen and extracted with 500 μL of 80% methanol for untargeted metabolomic profiling. The mixture was vortexed, incubated on ice for 5 min, and then centrifuged at 15,000× *g* for 20 min at 4 °C. The resulting supernatant was diluted with mass spectrometry-grade water to achieve a final methanol concentration of 53%, followed by a second centrifugation under the same conditions. The clarified supernatant was subsequently collected for LC-MS analysis [29]. Chromatographic separation was carried out on a Hypersil GOLD C18 column (Thermo Fisher Scientific, Runcorn, UK) maintained at 40 °C, with a flow rate of 0.2 mL min^−1^. For positive ionization mode, the mobile phases consisted of 0.1% formic acid and methanol, whereas for negative ionization mode, 5 mM ammonium acetate (pH 9.0) and methanol were used. Mass spectrometry data were acquired in both electrospray ionization modes over an m/z range of 100–1500, and MS/MS spectra were collected using a data-dependent acquisition strategy. The raw data files were processed using Compound Discoverer 3.1. Peak alignment and extraction were performed with a retention time tolerance of 0.2 min, a mass tolerance of 5 ppm, a signal intensity deviation of 30%, and a signal-to-noise threshold of 3. After removing features detected in blank samples, peak areas were normalized, and metabolites were annotated by comparison with mzCloud, mzVault, MassList, KEGG, HMDB, and LIPID MAPS databases using Compound Discoverer 3.1, Python 2.7.6, and R 3.4.3. Multivariate analyses were performed to characterize differences in muscle metabolomic profiles between the two culture systems. Principal component analysis (PCA) was used as an unsupervised approach to visualize overall variation among samples, whereas partial least-squares discriminant analysis (PLS-DA) and orthogonal partial least-squares discriminant analysis (OPLS-DA) were used to further evaluate group separation. PCA and PLS-DA were performed using metaX 1.4.2 [30], and differential metabolites were identified based on *t*-tests with criteria of VIP > 1, *p* < 0.05, and fold change ≥ 2 or FC ≤ 0.5.

### 2.5. 16S rRNA Sequencing in Intestinal Bacteria of M. salmoides

Microbial DNA was extracted from intestinal samples using the HiPure Soil DNA Kit (D3142, Magen, Guangzhou, China) according to the manufacturer’s instructions. The V3–V4 hypervariable region of the bacterial 16S rRNA gene was amplified using the primers 341F (5′-CCTACGGGNGGCWGCAG-3′) and 806R (5′-GGACTACHVGGGTATCTAAT-3′). PCR products were purified using AMPure XP beads (Beckman Coulter, Brea, CA, USA), quantified by real-time PCR, pooled in equimolar amounts and sequenced on an Illumina NovaSeq platform (Illumina, San Diego, CA, USA). Raw reads were quality-filtered to generate high-quality effective tags, which were clustered into operational taxonomic units (OTUs) at 97% sequence identity using the UPARSE algorithm (V7.0.1001) [31]. Taxonomic annotation of representative OTU sequences was performed using the Mothur classifier (V1.46.1) against the SILVA database (V138) [32]. Alpha diversity indices, including Chao1, Shannon and Simpson index, were calculated using QIIME (V1.9.1) [33]. Beta diversity analysis was performed in QIIME to evaluate differences in microbial community structure among samples. Similarity percentage analysis (SIMPER) was conducted using the vegan package in R (Version 2.15.3) to quantify the contribution of individual taxa to community differences between groups. Linear discriminant analysis effect size (LEfSe, V1.0) was further used to identify bacterial taxa with significant differential abundance and biological relevance between the two groups [34].

### 2.6. Statistical Analysis

Statistical analyses were performed using IBM SPSS Statistics 26.0 (IBM Corp., Armonk, NY, USA). Data are presented as mean ± standard error (SEM). Normality and homogeneity of variance were assessed using the Shapiro-Wilk and Levene tests, respectively. When these assumptions were not met, non-parametric tests were applied, including the Mann-Whitney U test for two-group comparisons. Differences in amino acid and fatty acid composition between groups were analyzed using independent-samples *t*-tests when appropriate. Unless otherwise stated, statistical significance was set at *p* < 0.05. For metabolomic data, multivariate analyses including PCA, PLS-DA, and OPLS-DA were performed as described in Section 2.4, and differential metabolites were screened using combined VIP, *p*-value, and fold-change criteria. Data visualization was performed using GraphPad Prism 11.0 (GraphPad Software, Boston, MA, USA) and Microsoft Excel 2024 (Microsoft Corp., Redmond, WA, USA).

## 3. Results

### 3.1. Growth Performance of M. salmoides Under Two Culture Modes

After 10 weeks of feeding with the commercial pelleted diet, largemouth bass reared in the rice-fish and pond culture systems showed comparable growth performance (Table 1). Final body weight (FW) did not differ significantly between the two groups, reaching 242.31 ± 66.20 g in the rice-fish system and 237.17 ± 58.76 g in the pond system (*p* > 0.05). Similarly, final body length was 21.13 ± 1.91 cm and 21.32 ± 1.80 cm in the rice-fish and pond groups, respectively, with no significant difference detected (*p* > 0.05). The specific growth rate (SGR) was also similar between culture systems, with values of 1.73 ± 0.05 in the rice-fish group and 1.70 ± 0.03 in the pond group (*p* > 0.05). In contrast, condition factor (CF) differed significantly between the two systems, with fish from the rice-fish system exhibiting a higher CF than those from the pond system (2.53 ± 0.44 versus 2.41 ± 0.34; *p* = 0.03). Overall, these results showed that largemouth bass maintained comparable growth performance under the two culture systems, although condition factor was significantly higher in the MR group.

### 3.2. Differences in Muscle Amino Acid Composition

As shown in Table S1, 17 amino acids were detected in the muscle tissue of largemouth bass, comprising eight essential amino acids and nine non-essential amino acids. No significant differences were observed between the two culture systems in the contents of total amino acids (TAAs), essential amino acids (EAAs) or non-essential amino acids (NEAAs). However, several free amino acids in largemouth bass muscle differed significantly between the two culture systems (Table 2). The levels of serine (Ser), valine (Val) and proline (Pro) were significantly lower in fish from the rice-fish culture system than in those from the pond system (*p* < 0.05). By contrast, alanine (Ala), cysteine (Cys) and lysine (Lys) were significantly higher in the rice-fish group (*p* < 0.01). These results indicate that the two culture systems were mainly associated with differences in individual free amino acids rather than the overall amino acid composition of muscle protein.

### 3.3. Differences in Muscle Fatty Acids Composition

Muscle fatty acid profiles differed markedly between culture systems (Table 3). Pond-cultured individuals (MP) showed substantially higher total fatty acid content than rice-field-cultured fish (MR) (2072.9 ± 148.48 versus 670.9 ± 53.89 mg 100 g^−1^, *p* < 0.01). The dominant fatty acids in both groups were C18:2n6c, C18:1n9c and C16:0, all of which were significantly more abundant in MP than in MR. Most other detected fatty acids, including C14:0, C16:1, C18:0, C18:3n3, C20:4n6, EPA and DHA, showed the same pattern, whereas C22:1n9 did not differ significantly between groups. At the class level, fish from pond had significantly higher contents of SFA, MUFA and PUFA, with PUFA representing the predominant fraction in both culture systems. Both n-3 and n-6 PUFA contents were higher in MP, but the n-3: n-6 ratio was significantly higher in MR than in MP (0.347 ± 0.04 versus 0.231 ± 0.01; *p* < 0.05). These results indicate that pond culture promoted overall fatty acid accumulation in *M. salmoides* muscle, whereas rice-field culture was associated with a more favorable n-3 PUFA balance.

### 3.4. Comparison of M. Salmoides Muscle Metabolomic Profiles in Two Culture Modes

Principal component analysis (PCA) revealed a clear separation in *M. salmoides* muscle metabolite profiles between MP and MR in both positive and negative ionization modes (Figure S1A,B). Consistently, partial least-squares discriminant analysis (PLS-DA) further distinguished the two culture systems, confirming culture mode-dependent metabolic divergence in both ionization modes (Figure S1C,D). Model validation indicated that the corresponding OPLS-DA models were reliable for discriminating muscle metabolic profiles between the two groups (Figure S1E,F). Untargeted metabolomic profiling identified 477 metabolites in positive ionization mode and 279 metabolites in negative ionization mode in muscle tissue. After 10 weeks of culture, 60 metabolites differed significantly in positive ionization mode, including 31 upregulated and 29 downregulated metabolites in the MP relative to the MR group (Figure 1A). In negative ionization mode, 67 differential metabolites were detected, comprising 34 upregulated and 33 downregulated metabolites (Figure 1B). KEGG enrichment analysis revealed distinct pathway-level differences between the two culture systems (Figure S2A,B). In positive ionization mode, the enriched pathways were mainly associated with amino acid biosynthesis, arginine and proline metabolism, glutathione metabolism, cysteine and methionine metabolism, and arginine biosynthesis. In negative ionization mode, the enriched pathways were primarily related to taurine and hypotaurine metabolism, purine metabolism, fatty acid biosynthesis, and cysteine and methionine metabolism. These differential metabolites were mainly associated with amino acid- and fatty acid-related metabolic pathways in largemouth bass muscle.

Specifically, in the positive ion mode, MP individuals showed notable alterations compared with MR, mainly involving amino acid, sulfur-containing amino acid, nucleotide, and lipid metabolism. Several amino acid-related metabolites showed higher abundance in MP, whereas guanidinoacetic acid and multiple acylcarnitines showed lower abundance, with the latter being associated with fatty acid transport and β-oxidation. Some lipid-related metabolites were also lower in MP group (Figure 1C). In the negative ion mode, differential metabolites were primarily associated with oxidized phospholipid metabolism, fatty acid oxidation, purine metabolism, and amino acid metabolism. Pond-cultured individuals exhibited increased levels of metabolites related to methylthioadenosine, purine, and amino acid metabolism. In contrast, oxidized phosphatidylcholines and lipid oxidation-related metabolites were generally reduced in MP samples, suggesting their relative enrichment in rice-fish system (Figure 1D). Overall, the differential muscle metabolites between the two groups were mainly associated with amino acid metabolism, purine metabolism, fatty acid transport and phospholipid-related processes.

### 3.5. Intestinal Microbiota Characteristics of M. Salmoides in Two Culture Modes

A total of 2078 operational taxonomic units (OTUs) were identified in the gut microbiota of largemouth bass. Of these, 273 OTUs were shared between the rice-fish and pond culture systems, whereas 1750 OTUs were unique to the rice-fish system and 55 OTUs were unique to the pond system. We next characterized the dominant microbial taxa by comparing the ten most abundant phyla and genera between the two culture systems. At the phylum level, the gut microbiota of MR individuals was dominated by Firmicutes (42.58%), Proteobacteria (29.62%) and Fusobacteriota (20.70%), whereas MP individuals were mainly characterized by Firmicutes (52.16%), Fusobacteriota (36.90%) and Proteobacteria (8.70%) (Figure 2A). At the genus level, the three most abundant taxa in MR were *Mycoplasma* (24.49%), *Cetobacterium* (20.53%) and *Clostridium_sensu_stricto_13* (10.35%), while MP was dominated by *Romboutsia* (38.68%), *Cetobacterium* (36.60%) and *Clostridium_sensu_stricto_1* (11.74%) (Figure 2B). Further comparison indicated that Desulfobacterota (*p* = 0.039) and *Romboutsia* (*p* = 0.022) were the major taxa differentiating the two culture systems at the phylum and genus levels, respectively. Intestinal microbial α-diversity analysis showed no significant differences in the Shannon or Simpson indices between the MR and MP groups. But the Chao1 (*p* = 0.031) and ACE indices (*p* = 0.031) differed significantly between two groups and were higher in MR than in MP, indicating greater microbial richness in rice-fish co-cultured fish. Principal coordinate analysis (PCoA) revealed a clear separation of gut microbial communities between the two culture systems. This pattern was further supported by ANOSIM analysis, which showed that between-group variation exceeded within-group variation (R = 0.426, *p* = 0.014). These results demonstrate significant differences in intestinal microbial community structure between the two culture systems.

LEfSe analysis further revealed significant differences in the gut microbiota composition of largemouth bass between the two culture systems (Figure 3A,B). The discriminative taxa enriched in the MR group were mainly affiliated with Mycoplasmataceae/*Mycoplasma* and Pseudomonadaceae/*Pseudomonas*, whereas the MP group was primarily characterized by enrichment of Peptostreptococcaceae/*Romboutsia*. These taxa therefore represented distinct microbial signatures associated with the two culture systems. Additionally, function prediction for intestinal microorganism found that membrane transport was significantly enriched in the gut microbiota of MP fish (*p* = 0.019), while the MR group showed significantly higher predicted capacities for amino acid metabolism, folding, sorting and degradation, biosynthesis of other secondary metabolites, aging and nervous system-related functions (*p* < 0.05). The predicted functional profiles of the intestinal microbiota also differed between the two groups.

## 4. Discussion

Our findings showed that largemouth bass reared in rice-field and pond culture systems had comparable overall growth performance but differed in muscle fatty acid composition, several free amino acids, metabolomic profiles and intestinal microbial communities. Notably, the clearest differences between the two systems were observed in edible muscle composition, particularly the quantity and proportional distribution of fatty acids. Previous studies have shown that integrated rice-fish farming can improve resource-use efficiency through nutrient cycling among rice plants, fish and environmental microorganisms, whereas pond culture generally provides more stable feeding conditions and greater production control [8,25]. Our findings further suggest that, under comparable feeding conditions, the effects of these two culture systems on largemouth bass were expressed primarily through quality-related traits and gut microecological features rather than overall growth performance.

### 4.1. Effects of Two Culture Systems on Growth Performance of M. salmoides

In terms of growth performance, final body weight, final body length and specific growth rate did not differ significantly between the different culture groups, suggesting that rice-field culture can sustain growth performance in largemouth bass at a level similar to conventional pond culture [5]. This result is relevant for production because rice-field systems generally have shallower water and more complex spatial structure than ponds. The comparable weight gain observed here indicates that rice-fish culture may be a feasible model for farming high-value carnivorous fish [4,35]. In addition, the significantly higher condition factor observed in the MR group further suggests that rice-field culture may affect body morphology or nutrient allocation in largemouth bass. Similar patterns have been reported in crayfish, where rice-field culture increased specific growth rate and condition factor relative to monoculture, with stable isotope analyses further showing that rice plants and aquatic vegetation can provide additional dietary resources in integrated farming systems [36]. Nevertheless, condition factor is a composite morphological index that can be affected by reproductive status, lipid deposition and feeding behaviour [37]. Further measurements of feed intake, hepatosomatic index, and directly determined total muscle lipid content are therefore needed to clarify whether the higher condition factor observed in the MR group reflects differences in overall lipid deposition, nutrient allocation, or body-shape variation. These findings therefore support the growth-related component of our first hypothesis, indicating that the contrasting culture environments did not substantially alter overall somatic growth during the experimental period, although body condition differed between the two groups.

### 4.2. Effects of Culture Systems on Muscle Amino Acid Composition

Hydrolyzed and free amino acids were analyzed to characterize complementary aspects of muscle amino acid composition. Hydrolyzed amino acids were determined to characterize the overall amino acid composition and nutritional quality of muscle protein, whereas free amino acids were analyzed to characterize the soluble amino acid pool, which is more directly associated with taste-related and metabolic characteristics of the edible muscle [35]. In this study, the total contents of hydrolyzed amino acids, essential amino acids and non-essential amino acids did not differ significantly between the two culture systems, suggesting that culture mode had limited effects on the overall amino acid profile and basal nutritional value of muscle protein in largemouth bass [38]. This stability may be attributable to the use of the same commercial formulated diet in both groups, indicating that muscle protein deposition was relatively conserved across culture environments during the experimental period [13]. By contrast, free amino acid composition appeared to be more responsive to culture system. These metabolites are associated with the taste characteristics of aquatic foods and are also involved in osmoregulation, nitrogen metabolism, antioxidant defense and energy metabolism [5,39,40]. Our study showed that alanine, cysteine, lysine and histidine were significantly enriched in the muscle of MR fish, whereas serine, valine and proline were more abundant in MP fish. Free alanine is generally regarded as an important sweetness-related amino acid in aquatic products [41]. Because alanine is generally associated with sweet taste, its higher concentration in MR muscle may contribute to differences in potential taste characteristics between the two groups. Cysteine is a sulfur-containing amino acid involved in glutathione synthesis and cellular redox processes, making its higher level in MR fish nutritionally and biochemically noteworthy [42]. The higher levels of serine, proline and valine in MP fish may also be relevant to potential taste differences, as serine and proline are commonly associated with sweet or mild taste attributes, whereas valine can contribute bitterness or taste complexity [43,44]. Together, these results indicate that the major amino acid differences between the two groups occurred in the free amino acid fraction rather than in the overall amino acid composition of muscle protein, potentially contributing to differences in taste-related characteristics. Direct sensory evaluation will be required in future studies to determine whether these compositional differences are perceptible to consumers.

### 4.3. Effects of Culture Systems on Muscle Fatty Acid Composition

Among the muscle quality traits examined, fatty acid composition showed one of the clearest responses to culture system. The individuals from MP group had significantly higher levels of total fatty acids, saturated fatty acids, monounsaturated fatty acids and polyunsaturated fatty acids than MR fish, indicating substantially higher absolute fatty acid contents in the edible muscle of MP fish. Consistent with this finding, previous studies have suggested that pond-reared fish may accumulate more muscle lipids under stable feeding conditions [5,12]. Differences in feeding conditions, activity and energy expenditure between culture environments have previously been proposed as factors associated with muscle lipid content in cultured fish [45]. The n-3/n-6 PUFA ratio is widely used to assess the nutritional balance of fatty acids in fish muscle, with higher values indicating a greater relative abundance of n-3 polyunsaturated fatty acids and a more favorable fatty acid composition [46]. In the present study, although MP individuals accumulated significantly higher absolute levels of total fatty acids, n-3 PUFA and n-6 PUFA, MR fish showed a higher n-3/n-6 PUFA ratio. From a dietary perspective, however, absolute fatty acid contents should be considered together with proportional indices. MP fish contained higher absolute levels of n-3 PUFA, including EPA and DHA, indicating that an equivalent portion of MP muscle would provide a greater amount of these nutritionally important fatty acids. In contrast, MR fish exhibited a higher n-3/n-6 PUFA ratio, reflecting a greater proportional contribution of n-3 relative to n-6 PUFA. Therefore, the two systems differed in both fatty acid quantity and proportional composition, rather than showing a simple nutritional advantage of one system over the other. From a production perspective, the higher n-3/n-6 PUFA ratio observed in MR fish, together with the comparable growth performance between the two systems, supports rice-field culture as a nutritionally distinctive and ecologically oriented alternative for largemouth bass farming.

### 4.4. Metabolomic Responses to Two Culture Modes

Untargeted metabolomics provided complementary biochemical evidence that the two culture systems were associated with different metabolic states in largemouth bass muscle [47]. Although the formulated diet and feeding regime were comparable between groups, the two culture systems differed markedly in habitat structure, water depth and environmental complexity. Such differences may alter fish activity, exposure to naturally occurring substrates and patterns of energy expenditure, thereby affecting substrate utilization and metabolic turnover in muscle [3,48]. These system-level differences provide a plausible biological context for the distinct amino-acid- and lipid-related metabolic profiles observed between MR and MP fish. KEGG pathway enrichment analysis showed that the differential metabolites were mainly associated with amino acid biosynthesis, taurine and hypotaurine metabolism, arginine and proline metabolism, glutathione metabolism and purine metabolism. Taurine and hypotaurine metabolism have been implicated in osmotic regulation and antioxidant defense [49], while arginine and proline metabolism are closely associated with nitrogen balance and stress adaptation in muscle [50]. Purine metabolism may further contribute to flavor formation through its links with umami-related nucleotide metabolism [51]. The differential metabolites identified in these pathways were associated with amino acid turnover, nitrogen metabolism, redox-related processes and nucleotide metabolism in muscle. Further analysis showed that several amino acid-related metabolites and methylthioadenosine-related metabolites were elevated in the MP group, these metabolites were mainly related to amino acid turnover, the methionine salvage pathway and sulfur-containing amino acid metabolism. In contrast, several acylcarnitines also differed between the groups. Because acylcarnitines participate in fatty acid transport and β-oxidation, these differences provide additional evidence of distinct lipid-related biochemical profiles in MP and MR muscle [52,53]. In negative ionization mode, oxidized phospholipids and lipid oxidation-related metabolites were relatively enriched in rice-field-cultured largemouth bass. These differences further indicate distinct phospholipid- and lipid oxidation-related metabolite profiles between the two culture systems. These metabolomic differences support the muscle metabolomics component of our second hypothesis, showing that MR and MP fish were characterized by distinct biochemical profiles, particularly for metabolites related to amino acid, nucleotide and lipid metabolism. Collectively, the metabolomic findings provide biochemical context for the observed differences in edible muscle composition, particularly the free-amino-acid and fatty-acid profiles. Rather than indicating a single regulatory mechanism, they suggest that the contrasting culture environments were associated with different patterns of amino acid and lipid metabolism in largemouth bass muscle.

### 4.5. Intestinal Microbiota Variations Responses to Two Culture Modes

The gut microbiota results further showed pronounced differences in intestinal microbial community structure between the two culture systems. The substantially greater number of unique OTUs, together with the higher Chao1 and ACE indices observed in MR group, indicates that MR fish exhibited greater gut microbial richness than MP fish. Consistently, a study of yellow catfish in a rice-fish co-culture system showed that gut microbial diversity and α-diversity indices increased significantly after rice flowering suggesting that environmental shifts within rice-fish systems may promote gut microbial diversity and richness [54]. In contrast, the absence of significant differences in the Shannon and Simpson indices suggested that overall gut microbial diversity and evenness were relatively stable between two systems. Culture system therefore appeared to influence the gut microbiota mainly by altering microbial richness and the abundance of specific taxa.

At the taxonomic level, the gut microbiota of rice-field-cultured fish was mainly enriched in taxa affiliated with Mycoplasmataceae/*Mycoplasma* and Pseudomonadaceae/*Pseudomonas*, whereas pond-cultured fish were primarily characterized by enrichment of Peptostreptococcaceae/*Romboutsia*. These taxonomic differences may have implications for intestinal physiology and fish health. *Mycoplasma* is frequently dominant in the intestine of healthy carnivorous fish, particularly salmonids [55]. Genome-resolved analyses have suggested that fish-associated *Mycoplasma* may form host-adapted symbiotic relationships and possess functions related to vitamin and amino acid biosynthesis and oxidative-stress protection [56]. More importantly, a recent study in Atlantic salmon found that *Mycoplasma* abundance was positively associated with the expression of intestinal barrier-related genes, suggesting a potential relationship with maintenance of gut barrier function [57]. The higher abundance of *Mycoplasma* in MR fish may therefore reflect a distinct host–microbiota interaction under rice-field conditions, although its health significance in largemouth bass requires direct validation. Certain *Pseudomonas* strains can enhance host defenses, for example, *Pseudomonas stutzeri* F2 increased innate immune enzyme activities, improved intestinal morphology, and enhanced resistance of spotted seabass to *Aeromonas veronii* [58]. However, other members of *Pseudomonas* are recognized as opportunistic pathogens in fish, and genus-level 16S data cannot distinguish beneficial from potentially pathogenic strains [59]. In contrast, the enrichment of *Romboutsia* in MP may be relevant to intestinal metabolic homeostasis. A *Romboutsia* strain isolated from tilapia was shown to ferment carbohydrates and produce short-chain fatty acids, suggesting a potential role in supporting intestinal energy metabolism [60]. Predicted microbial functions also differed between the two systems, with membrane transport-related functions more highly represented in MP fish and functions related to amino acid metabolism, protein folding and degradation, and secondary metabolite biosynthesis more highly represented in MR fish. Overall, the two culture systems appear to support distinct intestinal microbial configurations with potentially different implications for barrier function, immune regulation and disease resistance. Nevertheless, because the present study did not directly measure intestinal histology, immune responses or pathogen resistance, these health-related implications should be regarded as biologically plausible interpretations.

## 5. Conclusions

In summary, largemouth bass reared in rice-field and pond culture systems showed comparable growth performance but differed in several nutritional characteristics of edible muscle. MP fish contained higher absolute amounts of total quantified fatty acids, SFA, MUFA, PUFA, n-3 PUFA, EPA and DHA, whereas MR fish exhibited a higher n-3/n-6 PUFA ratio. Differences in free amino acids and muscle metabolomic profiles further indicated distinct amino-acid- and lipid-related biochemical states between the two culture systems. These results indicate that the two systems produce distinct nutritional profiles rather than a simple hierarchy of edible muscle quality. The intestinal microbiota also differed between MR and MP fish, reflecting distinct gut ecological responses to the two culture environments. Taken together, rice-field culture represents a promising ecological alternative for largemouth bass production because it maintained growth performance comparable to conventional pond culture while producing a nutritionally distinctive edible product. From a production perspective, the comparable growth performance suggests no evident biological production penalty for MR at the individual-fish level. However, a complete bioeconomic comparison would require additional data on total harvest biomass, feed efficiency, fillet yield, rice co-production, production costs and market returns. Future studies incorporating systematic environmental monitoring, sensory evaluation, physicochemical quality measurements, targeted lipidomics, bioeconomic assessment and additional seasonal or developmental sampling would further improve the comprehensive evaluation of largemouth bass produced under different culture systems.

## Figures and Tables

**Figure 1:**
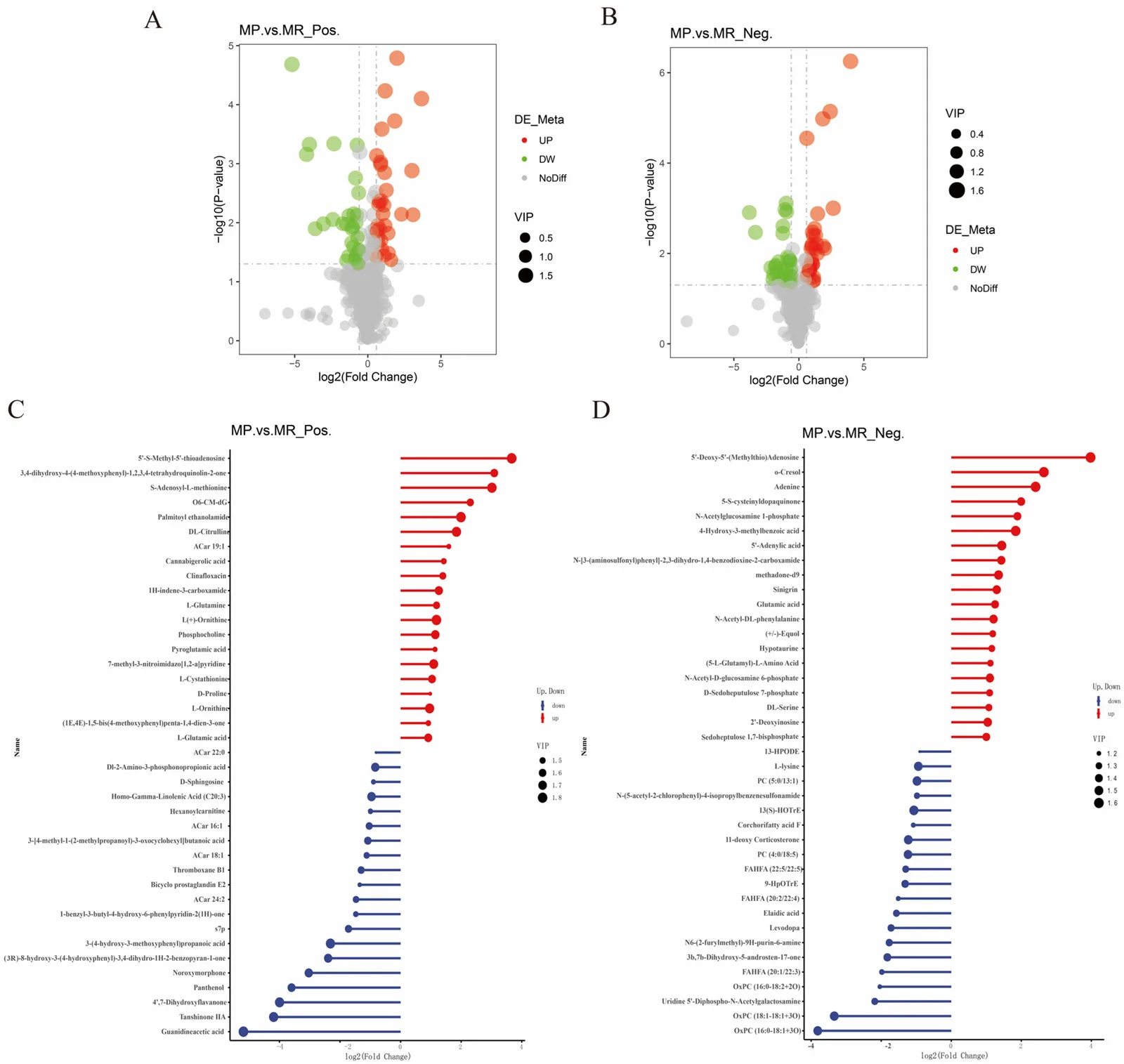
Different metabolites between pond farming (MP) and rice-fish farming (MR). (**A**,**B**), Volcano diagram for metabolites in positive and negative ion mode. (**C**,**D**), Differential metabolite lollipop plot in positive and negative ion mode. The length of each stem represents the magnitude of log_2_ (fold change), while the size of each point indicates the VIP value.

**Figure 2:**
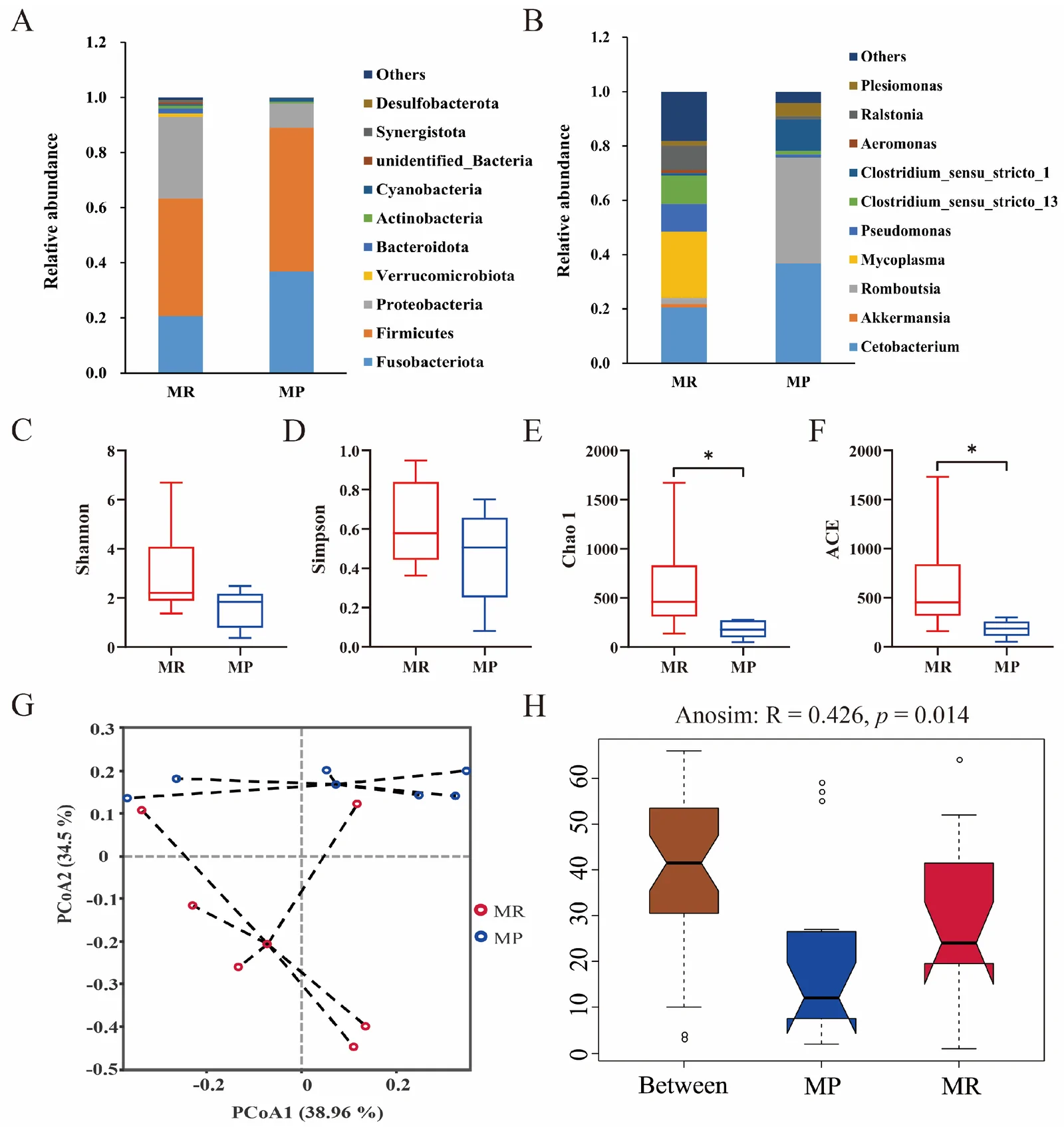
The proportions and diversity analysis of intestinal microbiota between two groups. (**A**,**B**), The top 10 phyla and genera of intestinal microbiota in the two groups. (**C**–**F**), Shannon index, Simpson’s index of diversity, Chao1 index and Abundance-based Coverage Estimator. (**G**), PCoA score plot. (**H**), Anosim test. * *p* < 0.05.

**Figure 3:**
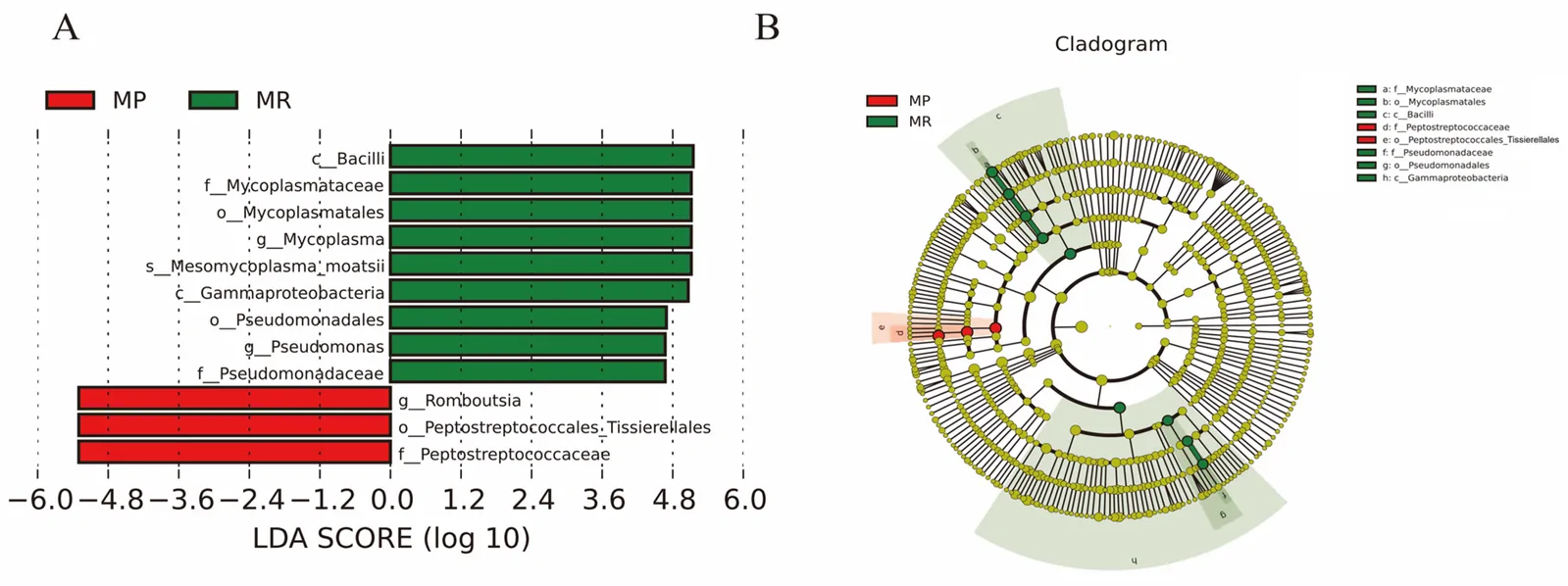
LEfSe analysis of differentially enriched intestinal microbiota in *M. salmoides* between two culture modes. (**A**) represents differentially enriched taxa identified by LEfSe analysis and their corresponding LDA scores. (**B**) presents the LEfSe cladogram, illustrating discriminative taxa enriched in each group across taxonomic levels from phylum to genus. Concentric circles represent taxonomic levels from phylum to genus, and node size is proportional to relative abundance. Yellow nodes indicate taxa without significant differences, whereas red and green nodes indicate biomarkers enriched in the corresponding groups. Taxa labelled with letters are identified in the legend.

**Table 1:** Growth performance of *M. salmoides*.

	MR	MP
Initial weight (g)	72.03 ± 10.32	72.1 ± 9.95
Initial length (cm)	14.99 ± 1.14	14.74 ± 0.75
Final weight (g)	242.31 ± 66.20	237.17 ± 58.76
Final length (cm)	21.13 ± 1.91	21.32 ± 1.80
SGR	1.73 ± 0.05	1.70 ± 0.03
CF	2.53 ± 0.44	2.41 ± 0.34

The data are expressed mean ± SD.

**Table 2:** The free amino acid composition in muscle of *M. salmoides* under two culture modes.

Amino Acids (mg/100 g, ww)	Groups		
	MP	MR	Sig.
Asp	4.0 ± 0.31	3.9 ± 0.19	ns
Thr	29.3 ± 1.11	28.5 ± 0.96	ns
Ser	10.3 ± 0.58	4.1 ± 0.09	0.001 **
Glu	19.3 ± 1.75	14.8 ± 0.85	ns
Gly	71.3 ± 4.42	65.5 ± 0.87	ns
Ala	30.0 ± 1.87	42.0 ± 2.04	0.005 **
Cys	1.2 ± 0.10	1.9 ± 0.07	0.001 **
Val	7.3 ± 0.23	5.7 ± 0.19	0.002 **
Ile	3.1 ± 0.14	3.4 ± 0.06	ns
Leu	16.0 ± 2.68	14.8 ± 1.65	ns
Lys	14.5 ± 3.00	38.5 ± 4.33	0.004 **
His	68.8 ± 1.80	56.3 ± 2.63	0.008 **
Pro	67.0 ± 3.08	53.3 ± 2.25	0.011 *
TFAA	341.9 ± 13.00	332.4 ± 11.25	ns

TFAA: Total Free Amino Acids, ns: not significant. Results were expressed as mean ± SE. Asterisks indicate significant differences, * *p* < 0.05 and ** *p* < 0.01.

**Table 3:** The hydrolyzed fatty acids composition in muscle of *M. salmoides* under two culture modes.

Fatty Acids (mg/100 g, ww)	Groups		
	MP	MR	Sig.
C14:0	27.7 ± 3.20	7.9 ± 1.30	0.001 **
C16:0	453.7 ± 35.77	151.3 ± 10.64	0.002 **
C16:1	50.2 ± 6.00	13.5 ± 2.24	0.005 **
C18:0	118.8 ± 4.56	45.0 ± 1.65	<0.001 **
C18:1n9c	591.3 ± 56.06	165.1 ± 23.75	<0.001 **
C18:2n6c	601.6 ± 39.81	185.1 ± 19.66	<0.001 **
C18:3n3	37.5 ± 3.02	10.5 ± 1.46	<0.001 **
C20:1	24.5 ± 1.93	8.5 ± 1.11	0.001 **
C20:2	21.3 ± 1.18	8.2 ± 0.63	<0.001 **
C20:3n6	9.7 ± 0.60	4.5 ± 0.13	0.002 **
C20:4n6	16.8 ± 0.53	8.2 ± 0.42	<0.001 **
C22:1n9	7.6 ± 0.84	6.2 ± 0.38	0.249
C20:5n3	8.9 ± 0.18	4.0 ± 0.12	<0.001 **
C24:1	6.4 ± 0.43	3.6 ± 0.07	0.003 **
C22:6n3	97.3 ± 3.17	52.8 ± 3.72	<0.001 **
Total SFA	600.2 ± 43.41	204.2 ± 13.24	0.002 **
Total MUFA	679.9 ± 63.07	194.4 ± 24.65	0.002 **
Total PUFA	792.9 ± 42.30	272.3 ± 16.79	<0.001 **
n-3 PUFA	143.6 ± 2.58	66.3 ± 3.27	<0.001 **
n-6 PUFA	628.0 ± 40.16	197.8 ± 19.32	<0.001 **
n-3: n-6 ratio	0.231 ± 0.01	0.347 ± 0.04	0.041 *
TFA	2072.9 ± 148.48	670.9 ± 53.89	0.001 **

SFA, saturated fatty acid; MUFA, monounsaturated fatty acid; PUFA, polyunsaturated fatty acid; TFA, total quantified fatty acids. Results were expressed as mean ± SE. Asterisks indicate significant differences, * *p* < 0.05 and ** *p* < 0.01.

## Data Availability

The original contributions presented in this study are included in the article/Supplementary Materials. Further inquiries can be directed to the corresponding authors.

## Supplementary Materials

The following supporting information can be downloaded at: https://www.mdpi.com/article/10.3390/foods15193369/s1. **Method S1:** Measurement of Growth Performance; **Table S1:** The hydrolyzed amino acid composition in muscle of *M. salmoides* under the rice-fish farming and the pond culture; **Figure S1:** The Multivariate statistical analysis for metabolomics data in muscle of *M. salmoides* under two culture modes; **Figure S2:** The KEGG enrichment analysis for different muscle metabolites between two culture modes; **Figure S3:** Function prediction for intestinal microorganism of *M. salmoides* farmed in rice field and pond.

## Abbreviations

The following abbreviations are used in this manuscript:

*M. salmoides*	*Micropterus salmoides*
FW	Final body weight
SGR	Specific growth rate
CF	Condition factor
TAA	Total amino acids
EAA	Essential amino acids
NEAA	Non-essential amino acids
FA	Fatty acid
SFA	Saturated fatty acids
MUFA	Monounsaturated fatty acids
PUFA	Polyunsaturated fatty acids
n-3 PUFA	n-3 polyunsaturated fatty acids
n-6 PUFA	n-6 polyunsaturated fatty acids
EPA	Eicosapentaenoic acid
DHA	Docosahexaenoic acid
PLS-DA	Partial least-squares discriminant analysis
OPLS-DA	Orthogonal partial least-squares discriminant analysis
VIP	Variable importance in projection
KEGG	Kyoto Encyclopedia of Genes and Genomes
HMDB	Human Metabolome Database
LIPID MAPS	Lipid Metabolites and Pathways Strategy
OTU	Operational taxonomic unit
UPARS	USEARCH-based sequence analysis pipeline
LEfSe	Linear discriminant analysis effect size
LDA	Linear discriminant analysis
PCoA	Principal coordinate analysis
ANOSIM	Analysis of similarities
MS-222	Tricaine methanesulfonate
RAS	Recirculating aquaculture system
