# Supplementary material for: Effects of Rice-Field and Pond Culture Systems on the Nutritional Quality, Metabolomic Profiles and Intestinal Microbiota of Largemouth Bass (Micropterus salmoides)
Source: Foods. 2026 Sep 22;15(19):3369. doi: 10.3390/foods15193369 (PMC13664413; doi:10.3390/foods15193369)
Supplement: Supplementary file 1 [file foods-15-03369-s001.zip › foods-4492242-supplementary.pdf]

Method S1. Measurement of Growth Performance

The growth performance was assessed by evaluating the following parameters: mean body weight, mean body length, condition factor (CF), weight gain rate (WGR) and specific growth rate (SGR).

$CF = 100 \times \text{body weight} / \text{body length}^3$
$SGR = 100 \times (\ln \text{average final weight} - \ln \text{average initial weight}) / \text{number of days}$

Table S1. The hydrolyzed amino acid composition in muscle of *M. salmoides* under the rice-fish farming and the pond culture

Amino acids (g/100g, ww)	Groups		
	MP	MR	Sig.
ASP	1.76 ± 0.079	1.85 ± 0.036	ns
Thr	0.78 ± 0.049	0.82 ± 0.018	ns
Ser	0.70 ± 0.018	0.69 ± 0.015	ns
Glu	2.56 ± 0.059	2.61 ± 0.060	ns
Gly	0.85 ± 0.024	0.84 ± 0.022	ns
Ala	1.06 ± 0.028	1.12 ±0.029	ns
Cys	0.14 ± 0.014	0.15 ± 0.017	ns
Val	0.82 ± 0.048	0.89 ± 0.016	ns
Met	0.40 ±0.056	0.45 ± 0.008	ns
Ile	0.72 ± 0.050	0.75 ± 0.018	ns
Leu	1.36 ± 0.060	1.43 ± 0.030	ns
Tyr	0.54 ± 0.035	0.56 ± 0.018	ns
Phe	0.65 ± 0.074	0.69 ± 0.019	ns
Lys	1.60 ± 0.044	1.66 ± 0.036	ns
His	0.42 ± 0.013	0.42 ± 0.005	ns
Arg	0.89 ± 0.072	0.90 ± 0.026	ns
Pro	0.60 ± 0.014	0.63 ± 0.023	ns
ΣEAA	7.64 ± 0.44	7.99 ± 0.17	ns
ΣNEAA	8.21 ± 0.21	8.44 ± 0.18	ns
ΣTAA	15.85 ± 0.63	16.43 ± 0.34	ns

EAA: Essential amino acid; NEAA: Non-essential amino acid; TAA: Total amino acid; ww: wet weight. The data are expressed mean ± SE, ns: no significant.

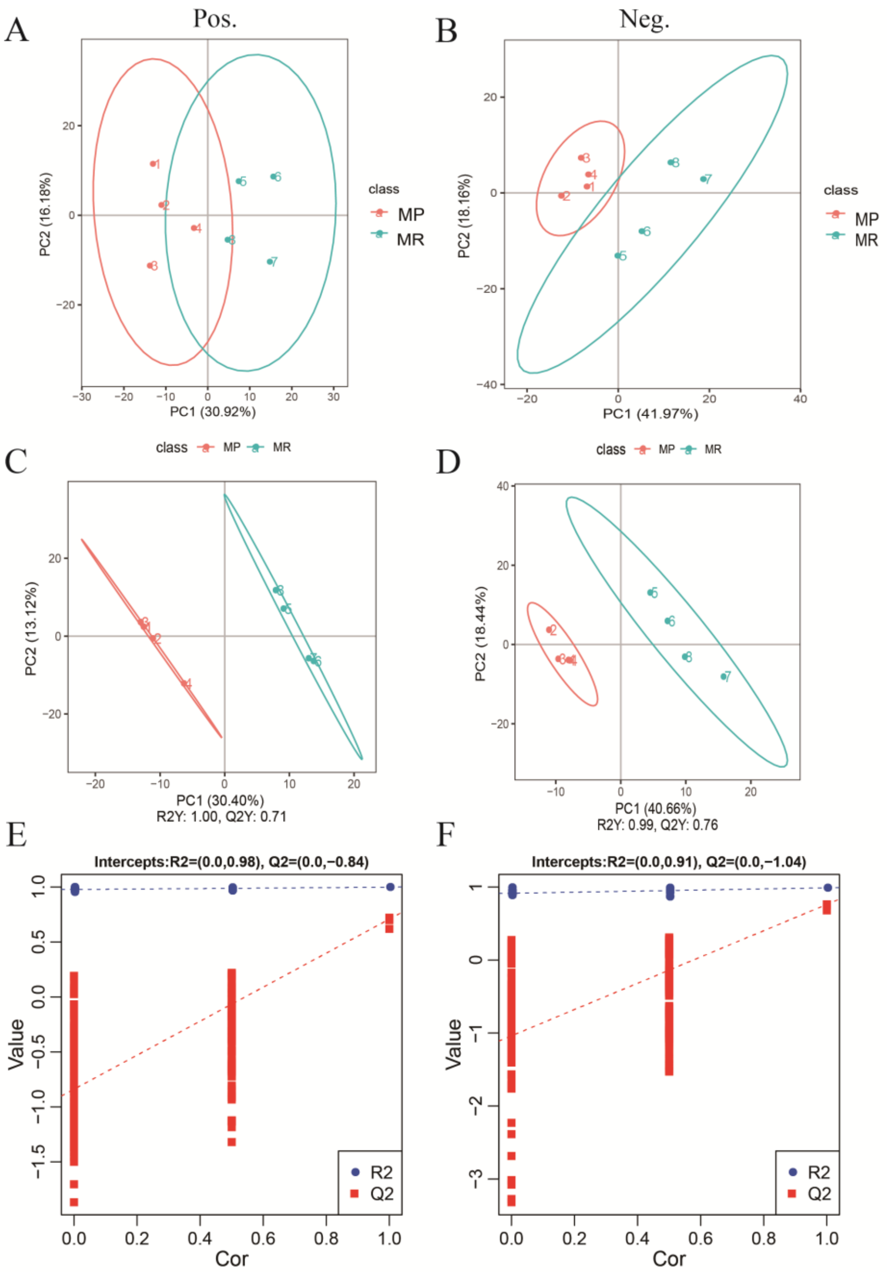

Figure S1. The Multivariate statistical analysis for metabolomics data in muscle of *M. salmoides* under two culture modes. A, PCA in positive ion mode. B, PCA in negative ion mode. C, PLSDA in positive ion mode. D, PLSDA in negative ion mode. E, Validation test in positive ion mode. F, Validation test in negative ion mode.

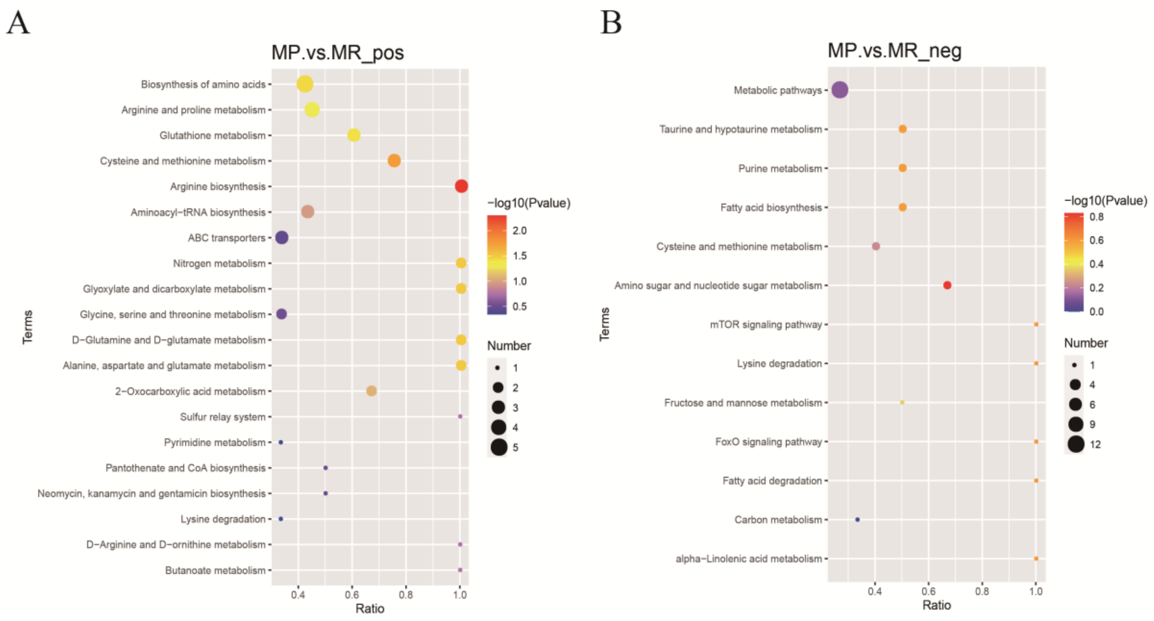

Figure S2. The KEGG enrichment analysis for different muscle metabolites between two culture modes. Two figures respectively represent pond farming (MP) and rice-fish co-culture (MR) under the positive ion mode (A) and negative ion mode (B).

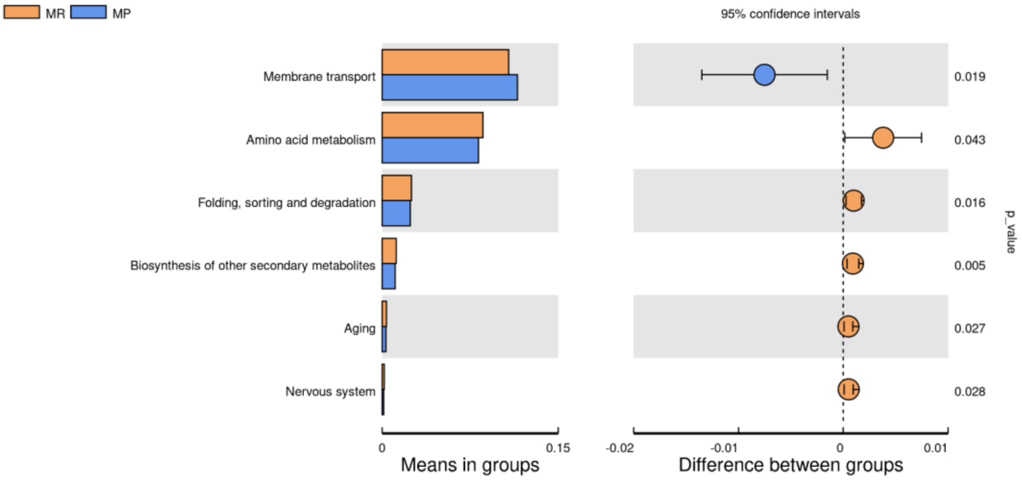

Figure S3. Function prediction for intestinal microorganism of *M. salmoides* farmed in rice field and pond.
